# Association among 2-min step test, functional level and diagnosis of dementia

**DOI:** 10.1590/1980-57642018dn13-010011

**Published:** 2019

**Authors:** Jessica Plácido, José Vinicius Ferreira, Felipe de Oliveira, Paula Sant’Anna, Renato Sobral Monteiro-Junior, Jerson Laks, Andrea C. Deslandes

**Affiliations:** 1 BSc, Laboratory of Neuroscience of exercise (LANeX), Institute of Psychiatry, Federal University of Rio de Janeiro (UFRJ), RJ, Brazil.; 2 MSc, Laboratory of Neuroscience of exercise (LaNEx), Institute of Psychiatry, Federal University of Rio de Janeiro (UFRJ), RJ, Brazil.; 3 BSc, Laboratory of Neuroscience of exercise (LaNEx), State University of Rio de Janeiro (UERJ), RJ, Brazil.; 4 BSc, Laboratory of Neuroscience of exercise (LaNEx), Institute of Psychiatry, Federal University of Rio de Janeiro (UFRJ), RJ, Brazil.; 5 PhD, Universidade Estadual de Montes Claros, Minas Gerais, Brazil.; 6 MD, PhD, Professor, Institute of Psychiatry, Federal University of Rio de Janeiro (UFRJ), RJ, Brazil.; 7 PhD, Professor, Laboratory of Neuroscience of exercise (LaNEx), Institute of Psychiatry, Federal University of Rio de Janeiro (UFRJ), RJ, Brazil.

**Keywords:** mild cognitive impairment, Alzheimer’s disease, aerobic capacity, cognitive function, physical function, comprometimento cognitivo leve, doença de Alzheimer, capacidade aeróbica, função cognitiva, função física

## Abstract

Aerobic capacity declines significantly throughout life, beginning at the age of 30 years and accelerating from 60 years, where a decline of 17% per decade is expected thereafter.

**Objective:**

To investigate the association between aerobic capacity and a diagnosis of mild cognitive impairment (MCI), mild AD or moderate AD in older adults, considering the risk classification of functional loss of the Step test.

**Methods:**

In this cross-sectional study, 93 patients (age >60 years) were evaluated (Healthy=36; MCI=18, AD=39). The step test was used to assess aerobic capacity, while overall cognitive status was measured using the MMSE. The groups were divided according to the risk classifications of functional loss into below or above the standard cut-off point for aerobic capacity.

**Results:**

Subjects in the functional loss risk group were approximately ten to fourteen times more likely to be diagnosed with mild (OR:10.7; p=0.001) or moderate (OR.=14.7; p=0.002) AD than their fitter counterparts. Low aerobic fitness was also associated with the MCI condition (OR=4.5; p=0.05), but only after controlling for educational level, age and sex. In the overall sample (N=93), there was an association between aerobic capacity and MMSE performance (R^2^=0.35; p<0.001) after controlling for confounding variables.

**Conclusion:**

low aerobic capacity was associated with cognitive decline, and older adults at risk of functional loss on the STEP test had greater chance of being diagnosed with MCI or AD after controlling for age, sex and education.

## RESUMO.

A capacidade aeróbica diminui significativamente ao longo da vida, começando com a idade de 30 anos e acelerando a partir dos 60 anos, onde se prevê um declínio de 17% por década.

**Objetivo:**

Investigar a associação entre a capacidade aeróbica medida e o risco de desenvolvimento de comprometimento cognitivo leve (CCL), DA leve e moderado em idosos, considerando as classificações de risco de perda funcional para o teste do STEP.

**Métodos:**

Nesse estudo de corte-transversal, foram avaliados 93 idosos (>60 anos) (saudáveis=36; CCL=18, DA=39). Para acessar a capacidade aeróbica foi utilizado o teste de STEP e o estado cognitivo global foi medido através do MEEM. Os grupos foram divididos em relação às classificações de risco de perda funcional em abaixo ou acima do ponto de corte padronizado para idosos.

**Resultados:**

Idosos incluídos no grupo de risco de perda funcional tinham aproximadamente dez a catorze vezes mais chances de serem diagnosticados com DA leve (O.R:10.7; p=0.001) e moderado (O.R.=14.7; p=0.002), do que os seus pares mais bem condicionados. Menores níveis de capacidade aeróbica também foram associados com o diagnostico de CCL (O.R=4.5; p=0.05), entretanto apenas após controle por idade, escolaridade e sexo. Na amostra total (N=93) houve uma associação entre a capacidade aeróbica e o MEEM (R^2^=0.35; p<0.001) depois de controlado pelas variáveis confundidoras.

**Conclusão:**

Idosos que se encontram dentro da classificação de risco de perda funcional medida através do teste de STEP possuem maiores chances de desenvolverem CCL, Alzheimer leve e moderado mesmo após controle por idade, sexo e escolaridade.

Aerobic capacity declines significantly throughout life, beginning at the age of 30 years and accelerating from 60 years, where a decline of 17% per decade is expected thereafter.^1^ It is believed that this decrease may intensify the process of aging and functional decline, contributing to greater dependence of elderly, through the impairment of their basic and instrumental (IADL) activities of daily living (ADLs).^2^

The maintenance of aerobic capacity during aging is also associated with a lower incidence of cardiovascular and metabolic diseases, preservation of cognitive performance^3^^,^ 4 and lower risk of dementia, such as dementia due to Alzheimer’s disease (AD).^5^ Since AD is a neurodegenerative disease and still has no cure, public health strategies have been focused on prevention and better clinical responses, such as preservation of cognitive ability and ADLs. The identification of cognitive decline and loss of functioning allows the implementation of early interventions and treatments, reducing the costs of disease and improving quality of life of patients.^6^ Considered an intermediate stage between healthy aging and dementia, mild cognitive impairment (MCI) represents a transitional phase in which patients present memory and/or executive function deficits, as well as reduced volume of brain structures common to AD.^7^ However, unlike individuals with Alzheimer’s, their independence for ADLs is still preserved.^8^ It is speculated that the rate of conversion from MCI to AD lies between 20 and 40%, being 10 to 15% per year.^9^

Lifestyle can help reduce the risk of conversion from MCI to Dementia. Recently, Müller and Chan^10^ demonstrated that, for every 1MET (*Metabolic Equivalent of Task*- approximately 3.5ml.kg^-1^.min^-1^) increase in VO_2peak_, there is an associated 8% decrease in risk of conversion to MCI and dementia. However, although ergospirometry was considered the “gold standard” method for obtaining the VO_2_ measurement, its use is limited by the high cost and need for equipment and specialists to perform the test, restricting its use to clinical environments and making it a technique of little ecological validity. An alternative is the STEP test from the *Senior fitness test* battery, proposed by Rikli and Jones.^11^ This evaluation is considered to have good reproducibility and easy applicability for assessing aerobic capacity in elderly people. In addition, its scores also allow the assessment of the risk of functional loss (RFL) of elderly related to aerobic capacity performance.

Therefore, the main objective of this study was to investigate the relationship between the level of aerobic capacity and the risk of diagnosis of MCI, mild and moderate AD in older adults. As a secondary objective, the correlation between aerobic capacity and global cognitive state was analyzed.

## METHODS

We recruited elderly (>60 years old), diagnosed with AD or MCI (according to criteria previously proposed by Petersen^12^) and cognitively intact, through medical staff at the Center of Alzheimer Disease of the Psychiatry Institute of the Federal University of Rio de Janeiro. Diagnostic assessment was performed by structured clinical interview SCID for assessment of mental disorders and according to the Diagnostic and Statistical Manual of Mental Disorders - Fourth Edition (DSM-IV). Patients were classified by the Clinical Dementia Rating (CDR): 0.5-1.0 (mild) 2.0 (moderate) and 3.0 (severe). The exclusion criteria for AD were: cerebral infarction; ongoing treatments such as electroconvulsive therapy and psychotherapy; any other presenting neurological disorders; as well as diagnosis of other types of dementia; and the presence of any physical disability that rendered the individual unable to perform assessments; and severe visual and/or hearing impairment. The exclusion criteria for healthy subjects were the presence of any physical disability that rendered the individual unable to perform assessments; the presence of neurological or mental disorders, and severe visual and/or hearing impairment. All patients signed the written informed consent form. This study was approved by the Research Ethics Committee of the IPUB-UFRJ, under registration permit CAAE:24904814.0.0000.5263 and was part of a larger research project entitled, “Efficacy of physical exercise in the treatment of Major Depression, Alzheimer’s Disease and Parkinson’s Disease”.

### Procedures and tests

The assessments were made in three visits. On the first day, participants signed the written informed consent form, underwent anthropometric measurements, completed an anamnesis, as well as the International Physical Activity Questionnaire (IPAQ-short version), Lawton Scale and MMSE. The second visit entailed a maximal treadmill test to predict VO_2max_ and to assess possible cardiac health problems. The STEP test was performed on the third visit.

### Step test

The 2-minute Step test is an easy-to-use approach for assessing aerobic capacity of older adults.^11^ A mark is made on a wall or door with tape at a height midway between the subject’s patella and iliac crest. After the signal “go”, the participant must alternate steps on the spot for the scheduled time period. The score is the number of times the right knee reaches the required height in 2 minutes and the cut-off point for risk of functional loss is 65 steps.

### VO_2_ protocol

The test consisted of a ramp protocol and a treadmill InbraMed Pro* was used.^13^ A fixed ramp protocol was used with determination of initial and final velocities and inclination. The increase in workload was progressive and continuous with gradual increments of velocity and/or inclination every 30 seconds. The assessment was programmed for patients to reach an estimated VO2max in 10 minutes, followed by a 6-minute recovery. A 12-lead Digital ECG electrocardiograph (MICROMED®) was used for monitoring and recording blood pressure and heart activity. Estimated VO2 was obtained using the following equation: VO_2_(ml/Kg/min) = velocity × [0.1+ (inclination/100 x 1.8)] + 3.5.^13^

### Statistical analysis

Normality and homoscedasticity of the sample were analyzed by Kolmogorov-Smirnov and Levene tests. Comparative analyzes were performed separating the groups by diagnosis (healthy, MCI, mild and moderate AD) and risk of functional loss (<65 Steps) using one-way ANOVA and the Kruskall-Wallis test according to the criteria of normality. To determine differences among groups, Bonferroni post hoc and Mann-Whitney tests were applied. An association between performance on the STEP test and on the MMSE was investigated using Spearman correlations for non-parametric data. The Chi-square analysis test was used to compare categorical variables. Associations between aerobic capacity and diagnosis were explored by multiple regression (hierarchical and stepwise) and logistic models. Statistical analysis was conducted using SPSS® software version 20.0 (IBM Corporation, New York, USA). The level of significance was p≤0.05

## RESULTS

**Figure 1:**
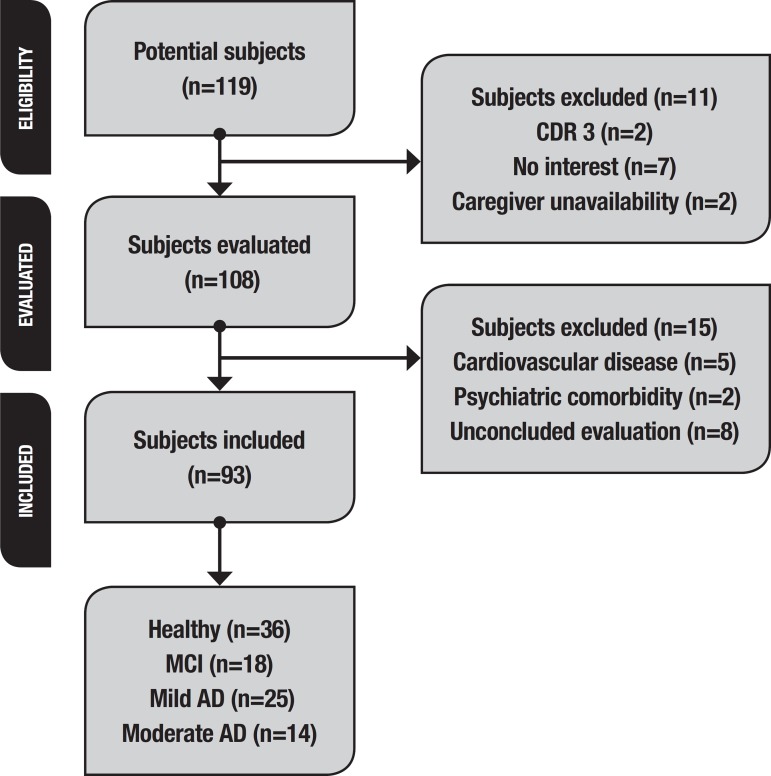
Flow chart of subject selection process.

Of the 119 participants initially recruited, 9 elders were excluded because they did not meet the study inclusion criteria, 15 participants had no interest in continuing the research and 2 dropped out due to unavailability of the caregiver (Figure 1). Thus, the final sample consisted of 93 participants, comprising 36 healthy elderly, 18 patients diagnosed with MCI, 25 with mild AD and 14 with moderate AD. Thirty-three elders (35.9%) were classified as having risk of functional loss on the STEP test (Healthy=14.3%, MCI=33.3%, mild AD=57.1%, moderate AD=56%). There were significant differences in age (p<0.02) and educational levels (p<0.02) among the groups. Also, groups varied signiﬁcantly for sex, where the majority of the study sample was female (p=0.03). The AD group was physically more classified as being at risk of functional loss, with mean STEP values of 63.8±27.2 reps (mild AD) and 54.6±26.5 reps (moderate AD), while means of healthy and MCI groups were 87.6±20.8 and 75.7±21.0 reps, respectively. These results were also observed for IADL evaluated by the Lawton Scale, which showed that the AD group was more dependent than the others. However, the groups did not differ in total physical activity levels, as measured by the IPAQ-MET (p=0.06 effect size=0.2). A significant difference was observed among the groups for cognitive performance measured by the MMSE (p≤0.001), showing that AD patients were more impaired than other participants. However, a Mann-Whitney analysis demonstrated no difference in overall cognitive state between healthy elderly and MCI subjects (p=0.40). After controlling for confounding variables, aerobic capacity (measured by STEP test) was found to be significantly associated with global cognition (R^2^=0.35 p<0.001), while VO_2_ max had a significant contribution to diagnosed condition (R^2^=0.19 p<0.001). Descriptive data of the sample is presented in Table 1. In addition, results of the multiple linear regression revealed that diagnoses can explain the decline in aerobic capacity evaluated by STEP, after controlling for age, sex and educational level (R^2^=0.45 p<0.001). Diagnosis alone represented 22% of the prediction of functional capacity (Table 2). Moreover, subjects in the functional loss risk group were approximately ten to fourteen times more likely to be diagnosed with mild (OR:10.7; p=0.001) or moderate (OR.=14.7; p=0.002) AD than their fitter counterparts (Table 3).

**Table 1:** Descriptive analyses of the samples.

		Healthy (n=36)	MCI (n=18)	Mild AD (n=25)	Moderate AD (n=14)	χ^2^/F	p
Age (years)		74.2±9.2	78.0±5.5	78.5±7.4	78.9±8.3	2.041^a^	0.02
Education (years)		12 (1-29)	15 (3-18)	11 (2-23)	12 (1-16)	6.025^b^	0.02
Sex (% of total cohort)	Female	30 (31.9%)	10 (11%)	16 (17.6%)	6 (6.6%)	8.743^b^	0.03
	Male	6 (6.6%)	8 (8.8%)	9 (8.8%)	8 (8.8%)		
Medication (n)		2 (0-9)	1 (0-7)	5 (1-11)	2.5 (1-9)	14.731^b^	0.002** ^++^
Chronic diseases (n)		0.5 (1-3)	1 (1-3)	2 (1-6)	0.5 (1-3)	14.398^b^	0.002** ^++^
Cardiovascular disease (n)		0 (0-1)	0.5 (0-2)	0 (0-2)	0 (0-1)	7.502^b^	0.05 ^||**^
BMI (kg/m^2^)		25.8 (20.9-34.0)	25.1 (22.0-32.2)	24.9 (18.7-38.2)	25.6 (18.6-28.9)	0.437^b^	0.93
VO_2_max (mL . kg^-1^.min^-1^)		22.2±6.2	21.1±4.7	18.2±4.6	17.7±6.5	3.626^a^	0.01**
STEP test (score)		87.6±20.8	75.7±21.0	63.8±27.2	54.6±26.5	8.553^a^	<0.001*
IPAQ (METs/week)		883.5 (33-12.558)	1221.7 (50-6.384)	462 (66-6.132)	772.5 (132-1.448)	7.382^b^	0.06
Lawton (score)		21 (6-27)	20 (11-21)	13 (8-18)	10 (7-17)	59.286^b^	<0.001* ^+^
MMSE (score)		29 (21-30)	29 (19-30)	21 (13-28)	15 (5-22)	59.040^b^	<0.001* ^+^

BMI: body mass index; MET: metabolic equivalent; MMSE: Mini-Mental State Examination; mean ± standard deviation; Median (minimum - maximum); χ^2^: chi-squared.

a one-way ANOVA;

b Kruskall-Wallis Test;

* Significant difference among Healthy, mild AD and moderate AD;

** Significant difference between Healthy and mild AD;

|| Significant difference between Healthy and MCI;

+ Significant difference among MCI, mild AD and moderate AD;

++ Significant difference between MCI and mild AD. Level of significance: p=0.05.

**Table 2:** Association between aerobic capacity and the different diagnoses.

Model		B	95% CI		p
R^2^=0.22*					
1	Diagnosis	-11.2	-15.6	-6.9	<0.001
R^2^= 0.45*					
2	Age	-0.5	-1.1	-0.04	0.03
	Sex	22.6	13.4	31.8	<0.001
	Education	0.6	-0.1	1.4	0.09
	Diagnosis	-12.0	-16.0	-8.0	<0.001

B: unstandardized coefficients. CI: confidence interval.

* level of significance: p ≤0.005.

**Table 3:** Association between Risk of Functional Loss (RFL) on STEP test and diagnosis of MCI and AD.

		MCI				Mild AD				Moderate AD		
		OR	(95% CI)	p		OR	(95% CI)	p		OR	(95% CI)	p
STEP test	Without RFL	Reference										
	RFL	3.0	0.76-11.71	0.11		7.6	2.22-26.20	0.001		8.0	1.93-33.10	0.004
	Without RFL*	Reference										
	RFL	4.5	0.99-21.10	0.05		10.7	2.65-43.64	0.001		14.7	2.63-82.11	0.002

OR: odds ratio; CI: confidence interval;

* Adjusted for age, sex and educational level. Level of significance: p ≤0.05.

## DISCUSSION

Aerobic capacity measured by the STEP test was positively correlated with global cognition. In addition, the performance classified as a risk for functional loss on the STEP test contributes to the increased chances of MCI and Alzheimer’s diagnosis in the mild and moderate stages of the disease. The different diagnoses explained 22% of the worsening of cardiorespiratory performance, represented by a decrease of about 11 repetitions on the STEP test. Some authors argue that this may represent a set of systemic, motor and cognitive changes that start in the pre-dementia stages and change as the disease progresses.^14^^,^ 15 Burns, Cronk et al.^15^ reported a reduction in VO_2peak_ among individuals with AD and its correlation with brain atrophy caused by dementia, suggesting the possibility that the metabolic dysfunctions occurring in the brain of these elderly are also occurring in the muscle periphery, making AD a systemic disease. Motor changes also demonstrate a worsening dependent on disease progression, especially for motor processes regulated by complex cortical mechanisms.^16^^,^ 17

When separated by RFL, physically vulnerable individuals were approximately 11 times more likely to be diagnosed with AD, and almost 15 times more likely when considering the influence of risk factors such as age, educational level and sex. Previous studies have shown a positive impact of aerobic capacity on cognition in elderly, indicating that cardiorespiratory resistance may protect this group from possible cognitive dysfunctions. Barnes, Yaffe et al.^18^ investigated the relationship of aerobic capacity in young elderly with cognitive function for 6 years. At the end of this period, individuals whose aerobic capacity was preserved had better cognitive performance, as evaluated by the MMSE. Voss, Weng et al.^19^ found that modifications in the neural pattern network (responsible for executive function and selective attention) during aging are mediated by cardiorespiratory capacity. According to the authors, these results seem to be independent of the level of usual physical activity. Aerobic capacity also seems to attenuate the relationship between important genetic risk factors and AD.^20^^,^ 21

After controlling for sex, age and educational level, regression analyses showed that elderly classified with RFL on the STEP test also have a risk of being diagnosed with MCI. Aerobic capacity has been shown to be an important neuroprotective factor during the disease course. Teixeira, Rezende et al.^22^ reported a relationship between the volume of the gray matter and the integrity of the white matter with the cardiorespiratory fitness of individuals with amnestic MCI, especially in frontal areas. These results are supported by Ding, Tarumi et al.^23^ who verified that high levels of aerobic capacity are correlated with the preservation of white matter integrity and the executive function of elderly with MCI, even after controlling for age, sex, lesion burden and disease progression, reinforcing the hypothesis of cognitive reserve. This neuroprotective effect may be associated with the influence of aerobic capacity on the vascular system through changes in cerebrovascular conductance and blood pressure.^24^

Although aerobic capacity is a variable strongly influenced by genetics (50%),^25^ physical exercise has shown to be an effective means of increasing it. Baker, Frank et al.^26^ found that aerobic exercise performed 4 times a week in elderly patients with MCI for 60 min (75%-85% of reserve heart rate) for 6 months was able to significantly increase VO_2max_ and VO_2_peak, as well as improve cognitive flexibility and attention. The authors found a relationship between VO_2_peak and executive function in the exercise group. Smith, Nielson et al.^27^ reported a 10% increase in the VO_2_peak of healthy patients and of those with MCI after 2 months with a walking intervention (50%-60% of reserve heart rate) 4 times a week, an increase followed by improvements in neural efficiency during a semantic memory test. Salisbury^28^ analyzed elderly with AD before and after completing 6 months of moderate aerobic training and found that each additional meter walked in the reevaluation in the *shuttle walk test* represented an improvement of 0.88 points on the ADAS-cog. Other studies have also demonstrated the efficacy of aerobic exercise in increasing aerobic capacity and cognition in elderly with MCI and AD,^29^^,^ 30 and likewise for multimodal training (aerobic, flexibility and strength).^31^ Strength training seems to improve aerobic capacity only in elderly whose relative VO_2max_ is less than 25ml.kg^-1^.min^-1^.^32^ However, strength training, regardless of the initial aerobic capacity, promotes increases in lower limb strength and mobility,^33^ which can help elderly people maintain functional and weekly physical activity levels.

These results appear to be mediated by a cognitive and vascular reserve. While cognitive reserve represents the efficiency of the damaged brain in maintaining its cognitive and functional capacity, through a compensation mechanism in its neural networks,^34^ vascular reserve is a specific competence of cerebral blood vessels to respond to increased metabolic demand and chemical, mechanical, or neural stimuli.^35^ Both abilities are dependent on the habits acquired by the individual during life and involve several mechanisms, such as cerebral blood flow, bioavailability of nitric oxide, production of reactive oxygen species (ROS) and trophic factors such as Brain-Derived Neurotrophic Factor (BDNF), Vascular Endothelial Growth Factor (VEGF) and Insulin-Like Growth Factor (IGF-1).^36^ However, during the course of aging, these systems are impaired, which tends to hinder these adaptive responses of the cerebral and vascular reserve.^35^ One of the physiological changes promoted by the increase in aerobic capacity is increased blood flow, not only peripheral but central, where this cardiovascular adjustment culminates in the regulation of the endothelial expression of nitric oxide, which in turn, performs BDNF and VEGF signalling.^35^ Other biochemical pathways involving ROS, trophic factors, and anti-inflammatory cytokines are also suggested as modulators of neuroplasticity induced by aerobic capacity and physical exercise.^37^^,^ 38

The present study has some limitations that must be considered, such as the small sample size and the estimated protocol used to assess VO_2max_. Moreover, there was no difference in the physical activity levels (measured by IPAQ) among groups. Although IPAQ is widely used and well validated, there are several limitations inherent to this questionnaire. Studies have reported a poor correlation between IPAQ and number of steps (measured by accelerometer) and also IPAQ overestimates physical activity in some populations.^39^^,^ 40 It is important to highlight that, in studies with dementia patients, the questionnaire is answered by the caregiver, which may contribute to some inconsistencies in the final score of the test. The cross-sectional design of the study precludes the establishment of a cause-effect relationship. Patients with MCI and AD may be more sedentary than healthy elderly due a decrease in the IADLs levels and leisure-time activities, but this sedentary behavior can also contribute to higher risk and low aerobic capacity in these individuals. In addition, information is lacking on the reliability and typical errors of the Step test in this specific population. However, unlike previous studies, the use of the STEP test allowed us to evaluate the aerobic capacity associated with the levels of physical independence and to understand how they may be related to the diagnosis of dementia. Furthermore, the STEP test is a simple and ecological evaluation, which facilitates the extension of these results for clinical use. Therefore, in community-dwelling elderly, aerobic capacity measured using the STEP test is positively correlated with overall cognition and associated with reduced risk of being diagnosed with MCI or AD. The evaluation of elderly aerobic capacity can contribute to the development of strategies to prevent dementia. Elderly people should be encouraged to practice physical exercises, mainly aerobic and multimodal, to help maintain cardiorespiratory levels above the functional risk score.
